# Evolutionary change in metabolic rate of *Daphnia pulicaria* following invasion by the predator *Bythotrephes longimanus*

**DOI:** 10.1002/ece3.9003

**Published:** 2022-06-05

**Authors:** Varsha Rani, Tim Burton, Matthew Walsh, Sigurd Einum

**Affiliations:** ^1^ 8018 Department of Biology Centre for Biodiversity Dynamics Norwegian University of Science and Technology Trondheim Norway; ^2^ Department of Community Ecology Center for Ecological Research Budapest Hungary; ^3^ 8019 Norwegian Institute for Nature Research Trondheim Norway; ^4^ 12329 Department of Biology University of Texas at Arlington Arlington Texas USA

**Keywords:** anti‐predatory behavior, evolutionary response, invasive species, metabolic rate, vertical migration

## Abstract

Metabolic rate is a trait that may evolve in response to the direct and indirect effects of predator‐induced mortality. Predators may indirectly alter selection by lowering prey densities and increasing resource availability or by intensifying resource limitation through changes in prey behavior (e.g., use of less productive areas). In the current study, we quantify the evolution of metabolic rate in the zooplankton *Daphnia pulicaria* following an invasive event by the predator *Bythotrephes longimanus* in Lake Mendota, Wisconsin, US. This invasion has been shown to dramatically impact *D*. *pulicaria*, causing a ~60% decline in their biomass. Using a resurrection ecology approach, we compared the metabolic rate of *D*. *pulicaria* clones originating prior to the *Bythotrephes* invasion with that of clones having evolved in the presence of *Bythotrephes*. We observed a 7.4% reduction in metabolic rate among post‐invasive clones compared to pre‐invasive clones and discuss the potential roles of direct and indirect selection in driving this change.

## Graphical abstract

Using a resurrection ecology approach, we compared the metabolic rate of *Daphnia pulicaria* clones originating prior to an invasion by the predator *Bythotrephes longimanus* with that of clones having evolved in the presence of *Bythotrephes*. We observed a 7.4% reduction in metabolic rate among post‐invasive clones compared to pre‐invasive clones. We discuss the potential roles of direct and indirect selection in driving this change.

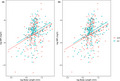

## 1 INTRODUCTION

Invasive predators are identified as one of several threats to global biodiversity. This makes it necessary to understand their potential lethal and non‐lethal impacts on native species, communities, and ecosystems (Gillis & Walsh, 2017; Lima, 1998; Pangle & Peacor, 2006). Invasion by non‐native predators may lead to decreased population abundance or complete extinction of native species (Havel et al., 2015). For example, a large number of cichlids went extinct after the introduction of Nile Perch into Lake Victoria (Goldschmidt et al., 1993). Furthermore, the ensuing predator–prey interactions can induce a range of evolutionary responses in native prey species (Barry & Syal, 2013; Krams et al., 2013) that include changes in patterns of movement (Bourdeau et al., 2013), changes in body size and reproductive effort (Fisk et al., 2007; Gillis & Walsh, 2017), increased ability to escape (O’Steen et al., 2002), or changes in feeding preferences (Singer et al., 1993).

Most of these studies have focused on phenotypic traits that are presumed to be under direct selection imposed by the invasive predator. Much less emphasis has been placed on understanding the *indirect* selective pressures induced by invasive predators. Among these indirect effects are shifts in selection caused by trophic cascades. Specifically, the invasive predators may reduce their prey abundance to such an extent that the prey is no longer resource limited (Walsh et al., 2016; White et al., 2006). An additional indirect effect of predators is that they can influence prey behavior, which may, in turn, further modify energy acquisition. For example, the presence of predators may cause prey to choose low‐quality habitats resulting in increased (rather than decreased) resource limitation under increased predator abundance (Lima & Dill, 1990). However, it remains largely unknown how invasive predators influence phenotypic evolution through such indirect effects, adding a hitherto unconsidered dimension to the nexus of evolutionary responses caused by invasive predators.

Given this potential for predators to not only exert direct selection, but to also alter the resource availability for the prey, traits that are involved in the conversion of energy may be of particular interest in this context. Metabolic rate (MR) is one such trait. It is the measure of rate for energy‐producing enzymatic reactions in a living being and thus has been connected to the rate of all biological activities (Brown et al., 2004). MR has been linked with key life‐history traits, such as growth, reproductive output, and survival (Pettersen et al., 2018). Furthermore, the broad‐sense evolvability in MR is similar in magnitude to other physiological and life‐history traits, pointing toward its evolutionary potential (Einum et al., 2019; Hansen et al., 2011). Mechanistically, a relationship between resource availability and MR can be hypothesized in terms of the "increased‐intake hypothesis" (Nilsson, 2002). A high resource availability allows a high intake, but with associated metabolic costs of maintaining high capacity in organs required for processing food. Conversely, if resource availability is low, paying this cost will not be beneficial and selection may favor reduced metabolic investment into such organs, and hence reduced MR. Indeed, studies have suggested that a relatively low MR might be favored in resource‐limited environments (reviewed by Burton et al., 2011). More recently, a study comparing guppy populations experiencing different predatory pressures shows a higher MR in guppies originating from high predation environment (Auer et al., 2018). However, in another comparative study of guppy populations, Handelsman et al. (2013) reported the opposite pattern. Finally, theoretical work indicates that the relationship between resource availability and optimal MR may be complex and that the optimal MR will be relatively higher at intermediate resource availability (Einum, 2014). Collectively, these studies suggest that the response of prey MR to increased predation pressure may be challenging to predict and that more empirical studies are required.

The freshwater zooplankton *Daphnia* has been used as a model organism for evolutionary research due to its substantially investigated life cycle and easy rearing under laboratory conditions (Lampert, 2006; Miner et al., 2012). In addition, *Daphnia's* ecology is well studied, as it is a key link in freshwater food webs, prey for fish, and other zooplanktivorous organisms, and highly efficient predator for phytoplankton and bacteria. It reproduces by parthenogenesis (i.e., clonally) under favorable conditions. This makes it a strong candidate for ecological and evolutionary studies involving changes in trophic control and related consequences (Stollewerk, 2010; Strecker & Arnott, 2008). Furthermore, *Daphnia* has been proven to be an ideal organism to use in so‐called “resurrection ecology” experiments. Under certain ecological conditions, *Daphnia* produces resting eggs as a result of sexual reproduction (also referred to as ephippia), some of which sink to the bottom of the water body and become embedded in the sediment where they can remain viable for years thereafter. The resting eggs within the ephippia can be hatched in the laboratory, and the resulting genotypes (each resting egg gives rise to a genetically unique individual, hereafter referred to as clones) kept in a common environment thus provide the means to observe phenotypic evolution among genotypes from vastly different time periods (Frisch et al., 2014; Landy et al., 2020).

Robison et al. (2018) reported predator‐induced phenotypic plasticity in MR in *Daphnia*, but beyond demonstration that it is a heritable trait (Einum et al., 2019), there is a lack of studies regarding the potential for evolutionary responses in MR. In the current study, we explore how MR evolves in *Daphnia pulicaria* from Lake Mendota, Wisconsin, USA. This lake has been invaded by the zooplanktivorous *Bythotrephes longimanus*, a zooplankton species native to Northern Europe (Hasnain & Arnott, 2019; Strecker et al., 2006). The main ecological impact caused by this invasion became evident in 2009, when a sharp decrease in the native population of *D*. *pulicaria* was observed (Walsh et al., 2016). Although possibly present prior to 2009, no *Bythotrephes* impacts on *Daphnia* had been detected previously. Invasion by *Bythotrephes* caused a rapid and substantial (~60%) decline in the biomass of *D*.* pulicaria*. This reduction in population density was accompanied by a one‐meter decrease in Secchi depth, a measurement indicating a substantial increase in surface water phytoplankton abundance (Walsh et al., 2016). Since phytoplankton is a key food source for *Daphnia*, one might expect that this leads to an increased resource availability, and with corresponding effects on the evolution of MR. However, this is complicated by the observation that the presence of *Bythothrephes* results in increased use of deeper, colder, and less productive parts of lakes among their zooplankton prey (Pangle et al., 2007), which may counteract the effect of increased food abundance in the surface layers. This, in addition to any direct selective pressures the predator may pose on this trait, makes it challenging to predict if and how MR may have responded to the invasion. Using a resurrection ecology approach, we compared the MR of *D*. *pulicaria* clones originating from prior to the *Bythotrephes* invasion with that of clones having evolved in the presence of *Bythotrephes*.

## 2 MATERIALS AND METHODS

### 2.1 Study species

Duplicate sediment cores were collected in February 2018 from Lake Mendota, Wisconsin, using a 1.5 m Griffith sediment corer with Livingstone drive rods, and live individuals were collected in June 2019. The cores were analyzed using ^210^Pb dating and loss‐on‐ignition assays to identify the demarcating depth for the pre‐ and post‐invasion period. For further details of resurrection methods, see Landy et al. (2020). For this study, 11 pre‐invasion and 11 post‐invasion clones were used. Based on the sediment core dating, the pre‐invasion clones that were used originated from the time period 1990 to 1997, whereas the post‐invasion clones were from 2015 to 2019. Hatched individuals from ephippia (representing all pre‐invasion and four post‐invasion clones, hatched during March 2019) and live‐collected individuals (representing post‐invasion individuals) were first kept at a 14 L:10D photoperiod at 16°C in 90 ml COMBO medium (Kilham et al., 1998) and fed non‐limiting supply of green algae (*Scenedesmus obliquus*, ~1.0 mg C/L/day). In December 2019, live individuals of each clone were transported to the Norwegian University of Science and Technology (NTNU). The selected clones were cultured and maintained at a population density of 10–15 individuals in 80 ml plastic jars filled with 50 ml selenium‐modified ADaM (Klüttgen et al., 1994, SeO_2_ concentration reduced by 50%) at a temperature of 17℃ and 16‐h photoperiod in Memmert Peltier incubators (Memmert GmbH). The medium was replaced every 2 weeks, at which point the position of jars within the cabinet was changed randomly. Each jar received Shellfish Diet 1800 (Reed Mariculture Inc) three times per week throughout the experimental duration at a cell concentration of 4 × 10^5^ cells/ml.

### 2.2 Metabolic rate measurement

We used the rate of oxygen consumption, i.e., respiration rate, to estimate MR (Barry & Syal, 2013; Fuhrman et al., 1961). Measurements were conducted during January‐June 2020 on animals that were fed and allowed to swim freely to approximate MR under field conditions. Oxygen consumption was measured at 17°C using a 24‐channel fluorescence‐based respirometry system (SDR SensorDish Reader, PreSens) placed in an incubator (Memmert Peltier‐cooled incubator IPP). The oxygen consumption rate was measured using a 24‐well microplate (capacity 200 µl, Loligo Systems) equipped with planar oxygen sensors. The wells were pre‐filled with air‐saturated ADaM before the transfer of individual *Daphnia* and were then sealed with an adhesive PCR film (Thermo Scientific). The effects of changing temperature on metabolic rate were minimized using a water bath at 17°C during the transfer of individuals into the wells and the sealing process. Oxygen concentration readings in the wells were taken every 3 min in darkness for 1.5–2 h using SDR version 38 software (PreSens). After the oxygen consumption measurement, all individuals were photographed under a stereomicroscope (Leica Microsystems GmbH) and measured digitally for body length (mm), from the tip of head crest to the point of tail spine attachment to the carapace, using IMAGEJ version 1.49v software (National Institutes of Health).

The experiment was conducted using only female individuals (*Daphnia* predominantly produce daughters by parthenogenesis under favorable conditions). To standardize feeding status, all individuals to be measured were kept in isolation in 50ml ADaM for 48 h prior to an experimental run, during which time they were fed twice. A total of 19 runs with 22 individuals (one in each well) per run were conducted, resulting in 418 measurements. However, 62 of these were excluded from statistical analyses due to individuals being injured during the process, or air bubbles being observed within the wells following measurements. Furthermore, to exclude data points where estimated oxygen consumption had high uncertainty, we only included data that had a strong linear decline in oxygen content during measurements. Thus, for each individual, we extracted the *r* ^2^ values from the fitted linear regressions between time since the start of the experiment and measured oxygen concentration, and excluded individuals (*n* = 8) for which *r* ^2^ < .95. In each run, two “blank” wells containing only ADaM were used as controls to estimate the level of microbial respiration, and the mean of this was subtracted from the observed oxygen consumption. Based on the volume of the wells, we calculated the amount of O_2_ (mg) at the start and end of the measurement for each individual, subtracted the microbial respiration, and divided by the duration of the measurement to obtain metabolic rate (mg/h). For further details on empirical methods and calculation of respiration rates, see Yashchenko et al. (2016).

### 2.3 Statistics

We used linear mixed‐effect modeling to model log_e_‐transformed MR as a response to log_e_ body length and clone type (pre‐ and post‐invasion) as fixed effects, and with run and clone line as random effects. Four different models with different fixed terms but the same random effects were fitted. The first model included the additive effects of body length and clone type. The second model contained both body length and clone type as well as their interaction. The third and fourth models used only body length and clone type as fixed effects, respectively.

Models were compared using Akaike Information Criterion (AIC) after fitting with maximum likelihood, while parameter estimates of the two best models were obtained by refitting the models with restricted maximum likelihood. The two models with the highest support were inspected for linearity, homoscedasticity, and normal distribution of residuals. All statistical analyses were done using R v. 4.0.2 (R Core Team, 2020), and linear mixed‐effect models were fitted using the *lmer* function in the *lme4* package (Bates et al., 2015).

## 3 RESULTS

**FIGURE 1:**
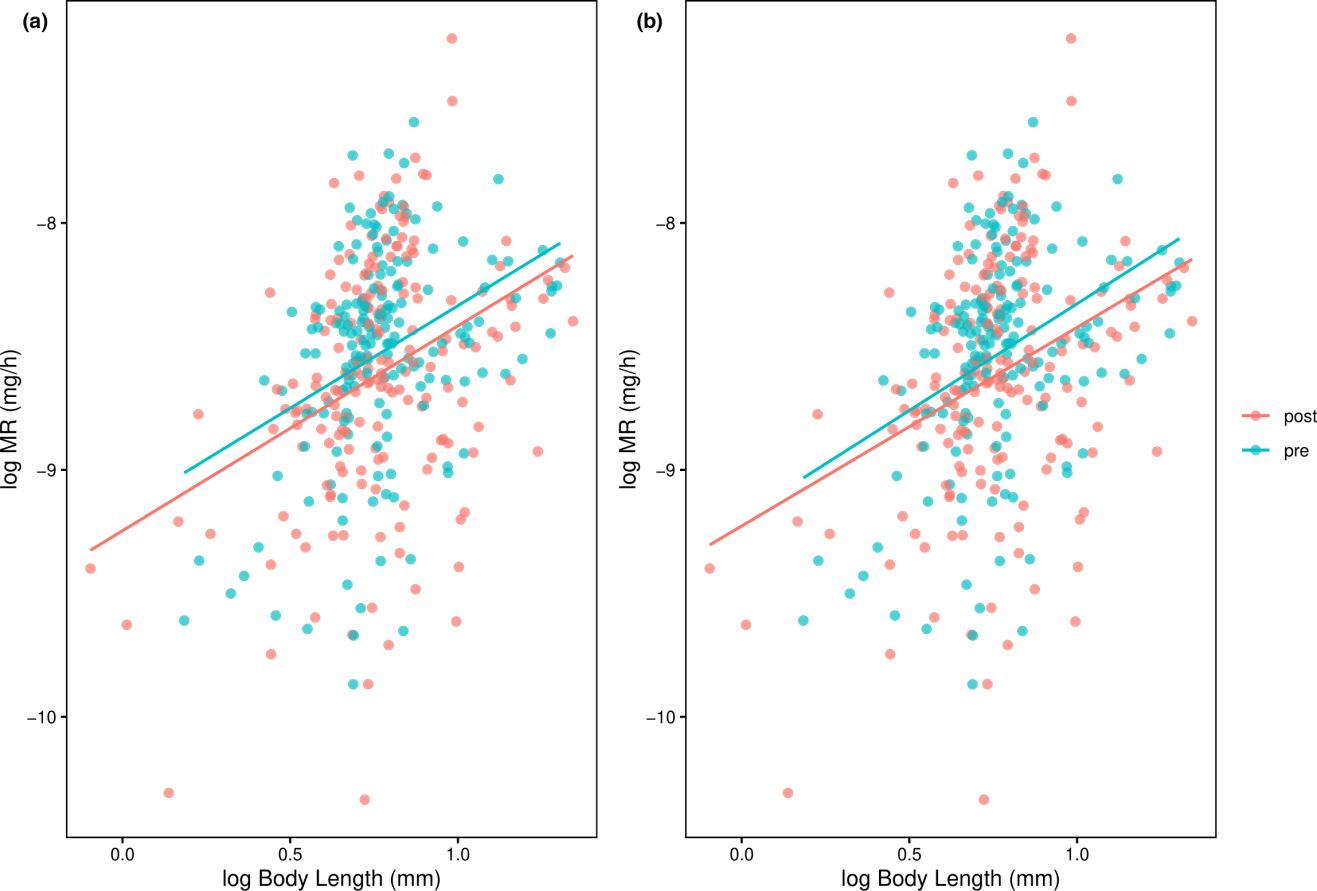
Relationship between log metabolic rate (MR) and log body length for *Daphnia pulicaria* originating from the pre‐ and post‐invasion periods by the predatory *Bythotrephes longimanus*. Lines give predictions for the two types of clones from (a) an additive model and (b) a model including an interaction between clone type and body length

The two models that contained effects of both body length and clone type received considerably stronger support than those containing only one of these terms (Table 1). Support for these two top models was similar in magnitude. For the model with only additive effects, MR increased with body length, and post‐invasion clones had a lower MR than pre‐invasion clones (Figure 1a). For the model that included the interaction, the interaction term was relatively weak, with the 95% CI including zero (Table 2), and the predicted MR for post‐invasion clones was lower than that for the pre‐invasion clones across the whole range of body lengths (Figure 1b). For the model with only additive effects, the predicted MR of an individual of the mean body length (2.2 mm) was 9.01 × 10^−5^ mg/h and 8.34 × 10^−5^ mg/h for pre‐ and post‐invasive clones, respectively. This corresponds to a reduction of 7.4% in MR of the post‐invasive clones compared to the pre‐invasive clones.

**TABLE 1:** AICc comparisons of candidate models explaining variation in metabolic rate in *Daphnia pulicaria* as a function of body length and clone type (pre‐ and post‐invasion by the predatory *Bythotrephes longimanus*)

Models	Fixed variables	K	AIC_C_	ΔAIC_C_	w_i_	acc w_i_
1	Body length + clone type	6	303.23	0	0.48	0.48
2	Body length × clone type	7	303.48	0.24	0.43	0.9
3	Body length	5	306.58	3.35	0.09	1.0
4	Clone type	5	451.11	147.88	3.73 × 10^−33^	1.0

Note

Measurement run and clone line were included as random intercept in all models.

Abbreviations: K, number of parameters (K‐2) in model; ΔAIC_C_, difference in AIC_C_ values between the given model and best‐fitting model among the candidates; w_i_, AIC_C_ weight; acc w_i_, sum of AIC_C_ weights.

**TABLE 2:** Summary of the best‐fitting linear mixed‐effect model estimating the effects of body length and clone type (pre‐ and post‐invasion by the predatory *Bythotrephes longimanus*) on metabolic rate in *D pulicaria*

	Estimates	95% CI
Additive		
Intercept	−10.02	−10.27 to −9.78
Log (body length)	1.83	1.60 to 2.07
Clone type (pre‐invasive)	0.08	0.01 to 0.15
Interaction		
Intercept	−9.95	−10.22 to −9.69
Log (body length)	1.75	1.47 to 2.02
Clone type (pre‐invasive)	−0.11	−0.39 to 0.18
Log (body length): Clone type (pre‐invasive)	0.25	−0.11 to 0.61

Note

“Additive” denotes the best model, whereas “Interaction” denotes the second‐best model in Table 1.

## 4 DISCUSSION

In this study, we tested if the invasion of a non‐native predator is associated with an evolutionary change in the metabolic rate (MR) of its prey species. We used *Daphnia pulicaria* clones from Lake Mendota, USA, as the study prey species since this lake has been documented to have an established population of the non‐native predator *Bythotrephes longimanus* that have caused large cascading effects on the pelagic ecosystem of this lake (Walsh et al., 2016). A resurrection ecology approach enabled us to rear pre‐ and post‐invasion clones in the lab and to compare their MR under highly controlled experimental conditions. Our results demonstrate an evolutionary change in MR among post‐invasive clones, supporting the view that the predation regime influences the optimality of this trait. Specifically, post‐invasion clones had a reduced MR compared to pre‐invasion clones.

The observed evolutionary changes may be caused by either direct or indirect selective pressures imposed by the predator invasion. For direct selection, the observed evolutionary change would require a higher predation rate on genotypes with a higher MR. This could occur if high MR genotypes are more active and as a result more frequently encounter predators. Alternatively, MR could be positively genetically correlated with traits that increase susceptibility to predation. However, when comparing pre‐ and post‐invasion clones of the same species from the same lake, Landy et al. (2020) did not find any significant evolutionary changes in the horizontal migration index (a measure of activity), nor in the life‐history traits that they quantified (age at maturation, size at maturation, fecundity). They did find a pronounced reduction in positive phototaxis in post‐invasion compared to pre‐invasion clones. This is likely linked to selection for diel vertical migration (DVM) in the presence of the visually hunting *Bythotrephes* (Pangle & Peacor, 2009; Strecker & Arnott, 2008). Under DVM, individuals move to deeper waters at dawn and return toward the surface at night, and this behavior has a genetic component (Hasnain & Arnott, 2019; Rozenberg et al., 2015). One might speculate that this behavior is genetically correlated with MR. However, if this is the case, it may seem most likely that these traits would be positively correlated. Selection for DVM in post‐invasive clones would then be expected to result in a correlated increase rather than a decrease in MR. Direct selection by the predator may therefore be less likely to drive the observed evolutionary decline in MR.

An alternative mechanism behind the observed evolutionary change is that even if the invasive predator has caused an increase in productivity of surface waters, the *Daphnia* do not benefit from this due to the shift in their vertical distribution associated with DVM. Among genotypes that undertake such habitat shifts and successfully avoid predation, a new selective regime will be experienced, as they spend considerable amounts of time in the deeper water layers which receive less light and are less productive in terms of phytoplankton abundance. Under such an “ecology of fear” scenario (Zanette & Clinchy, 2019), the novel predator may in fact not cause a release of food restriction. Rather, the behavioral escape by the prey may create a stronger food limitation which may in turn select for a reduced MR (Burton et al., 2011). However, even this mechanism may not be straightforward to predict. First, the deeper and less productive layers of lakes are also colder, which reduces the MR (Gillooly et al., 2001) and hence the metabolic demand for resources. Second, the *Daphnia* may compensate for the reduced food intake during daytime by increasing their food intake during night when they move up into the productive surface layers (but see Lampert, 1989 for a critical review of such compensation in *Daphnia*). To evaluate the likelihood of such a mechanism, further efforts should be made to understand how *Bythotrephes* shape the seasonal dynamics of energetic constraints in *D*. *pulicaria*, for example, through studies of size‐specific fecundity (Lampert, 1978) or lipid contents (Tessier & Goulden, 1982).

Although we focus on the potential role of changing predatory patterns, Lake Mendota has for more than a century been heavily influenced by human activity, including wastewater discharge and agricultural and urban runoff leading to pronounced eutrophication (Lathrop, 2007). Thus, we cannot rule out that changes in the selective regimes associated with changing environmental conditions aside from the change in predation level can have caused the observed evolutionary changes. However, these other influences have a long history that predates the time of origin for the clones used in the current study (1990–2018), and thus it is not clear that these would result in a consistent temporal change in selection regimes. Data on nutrient inputs and concentrations in this lake have been highly variable among years without showing clear temporal trends at least since the 1970s (Lathrop & Carpenter, 2014).

The majority of previous studies on the role of predators in shaping MR of their prey have focused on phenotypically plastic responses. These studies have shown that the extent and direction of change in metabolic rate in response to predators are highly species‐specific, and even context specific. For example, tadpoles may increase their MR during short‐term exposure to a predator, whereas the long‐term response is a reduction in MR, presumably to compensate for the behavioral reduction in feeding activity (Barry & Syal, 2013; Steiner & Buskirk, 2009). Damselflies and amphipods increase their MR in response to predatory cues (Gjoni et al., 2020; Slos & Stoks, 2008), as does *Daphnia magna* in some studies (Beckerman et al., 2007), but not in others (Pauwels et al., 2010; Stibor & Machacek, 1998). Lobsters decrease their MR when exposed to predators (Briceño et al., 2018). If such variable plastic responses are adaptive, it is perhaps not surprising that similar differences exist in terms of evolutionary responses across species and systems. Strikingly, even separate populations within a single species (guppies) show opposite evolutionary trajectories of MR in response to a change in predation regime (Handelsman et al., 2013 vs. Auer et al., 2018). The current study adds to the limited knowledge on evolutionary responses of MR to a change in predator exposure. We tentatively conclude that the response observed in the present study is not a response to selective predation. Rather, it may be an outcome of a change in the resource limitation. However, we emphasize that there is a need for more studies across populations and species, in combination with controlled experimental studies, to obtain a more mechanistic and better predictive understanding.

## AUTHOR CONTRIBUTIONS

**Varsha Rani:** Conceptualization (equal); Formal analysis (lead); Investigation (lead); Methodology (equal); Writing – original draft (lead); Writing – review & editing (equal). **Matthew Walsh:** Conceptualization (supporting); Resources (equal); Writing – review & editing (equal). **Tim Burton:** Conceptualization (equal); Formal analysis (supporting); Resources (equal); Writing – review & editing (equal). **Sigurd Einum:** Conceptualization (equal); Formal analysis (supporting); Methodology (equal); Project administration (lead); Resources (equal); Writing – review & editing (equal).

## CONFLICT OF INTEREST

The authors declare no conflict of interest.

## Data Availability

The data used to draw graphs and tables in the study is available on Dryad https://doi.org/10.5061/dryad.95x69p8nc.
